# Hereditary Elliptocytosis Resulting From Heterozygosity for β Spectrin Tandil

**DOI:** 10.1002/ajh.27744

**Published:** 2025-06-18

**Authors:** María‐Angustias Molina‐Arrebola, Barbara J. Bain

**Affiliations:** ^1^ Unidad de Hematología y Hemoterapia, Área de Biotecnología Hospital Universitario Poniente El Ejido Almería Spain; ^2^ Centre for Haematology St Mary's Hospital Campus of Imperial College London Faculty of Medicine London UK

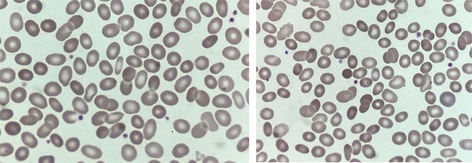

A 57‐year‐old Spanish woman was referred for investigation of chronic anemia. Her mother and grandmother also had a history of anemia. There was no jaundice and no abdominal pain or tenderness, but abdominal ultrasonography showed homogeneous splenomegaly, the spleen measuring 14.2 cm. She had required a transfusion on one occasion when the hemoglobin concentration (Hb) fell to 71 g/L. Her blood count showed Hb 130 g/L, mean cell volume 76.8 fL, mean cell hemoglobin 25.9 pg, and mean cell hemoglobin concentration 33.7 g/L. The reticulocyte count was 197 × 10^9^/L. Bilirubin was normal at 0.72 mg/dL, and lactate dehydrogenase was normal at 229 U/L. Haptoglobin was undetectable. Serum ferritin was increased to 411 ng/mL. Flow cytometry‐based eosin‐5‐maleimide (EMA) binding was low at 0.81 (normal > 0.86). Examination of a blood film showed elliptocytes, but the findings were not typical of hereditary elliptocytosis; in addition to elliptocytes, there were ovalocytes and some spherocytes (images, ×100 objective). Molecular analysis was therefore undertaken. Next‐generation sequencing showed heterogeneity for β spectrin Tandil, a pathogenic genetic variant, NM_001355436.2:c.6124_6130del p.(Asp2042Trpfs*33) in the β spectrin gene (*SPTB*) with a 7‐nucleotide deletion that causes a frameshift, generating a premature stop codon at position 33 of the new reading frame.

β spectrin Tandil was first described in a South American Indian family in Argentina. The hematologic abnormality resulting from this mutation was designated hereditary elliptocytosis, although blood films showed more ovalocytes than are usual in hereditary elliptocytosis, and ektacytometry indicated a spherocytic component [1]. Similar observations have been made with other truncated β spectrin variants, including β spectrin Rouen [2], β spectrin Le Puy [2], and β spectrin Prague [3]. The designation spherocytic elliptocytosis has sometimes been used [3]. Most cases of hereditary elliptocytosis result from mutation of the α spectrin gene (*SPTA1*) or of *EPB41* encoding protein 4.1. A minority result from a β spectrin mutation, including some cases with truncated β spectrin and atypical morphology.

## Conflicts of Interest

The authors declare no conflicts of interest.

## Data Availability

The authors have nothing to report.
